# Environmental Assessment and Eco-Efficiency of Airport Pavements Incorporating Warm RAP Base Layers

**DOI:** 10.3390/ma19091794

**Published:** 2026-04-28

**Authors:** Washington Camatari Junior, Tales Ribeiro Santos, Vinicius Storto Martinez Senra, Matheus Assis Maia, Filipe Almeida Corrêa do Nascimento, Antônio Carlos Rodrigues Guimarães, Sergio Neves Monteiro, Lisley Madeira Coelho

**Affiliations:** 1 Department of Fortification and Construction, Military Institute of Engineering-IME, Praça General Tibúrcio, 80, Urca, Rio de Janeiro 22290-270, Brazil; wscamatari@gmail.com (W.C.J.); tales.riba@ime.eb.br (T.R.S.); vinicius.storto@ime.eb.br (V.S.M.S.); assis.matheus@ime.eb.br (M.A.M.); filipe.nascimento@ime.eb.br (F.A.C.d.N.); guimaraes@ime.eb.br (A.C.R.G.); 2 Department of Materials Science, Military Institute of Engineering-IME, Praça General Tibúrcio, 80, Urca, Rio de Janeiro 22290-270, Brazil; snevesmonteiro@gmail.com

**Keywords:** life cycle assessment, reclaimed asphalt pavement, eco-efficiency, airport pavements

## Abstract

Strategies based on the use of recycled materials have been widely discussed as alternatives to reduce environmental impacts in transport infrastructure. In pavement engineering, the use of Reclaimed Asphalt Pavement (RAP) in base layers offers environmental benefits; however, its benefits depend on processing conditions and structural performance. Chemical stabilization techniques, although mechanically effective, tend to introduce environmental hotspots associated with binder production. In this study, controlled thermal conditioning of RAP is evaluated as a warm base solution without chemical stabilizers in the context of airport pavements. A comparative life cycle assessment was conducted under a production- and construction-stage scope (A1–A3 and A5, excluding transportation under equivalent logistical assumptions), considering untreated RAP, heated RAP, and RAP stabilized with emulsion and cement, and was integrated with mechanistic–empirical structural performance analyses. The results indicate that, although heated RAP presents intermediate absolute environmental impacts due to additional energy consumption, it achieves the highest eco-efficiency, expressed as the lowest ratio between global warming potential (IPCC 2023) and estimated structural service life. In the analyzed scenarios, the warm base showed approximately 71% lower environmental impact per year of service than untreated RAP and about 90% lower than the emulsion-stabilized alternative. These findings suggest that performance-based sustainability assessment can reveal environmental advantages in solutions that exhibit moderate increases in production-stage impacts but enhanced structural longevity. It should be noted that the conclusions are conditioned by the adopted production and construction system boundaries, which do not include the use, rehabilitation, or end-of-life phases.

## 1. Introduction

The incorporation of recycled materials and the adoption of construction processes at moderate temperatures (100–150 °C) have been proposed as promising ways to reduce the environmental impacts of transportation infrastructure and, simultaneously, to increase the value of waste materials generated during construction [1]. However, the environmental benefits associated with these approaches are not automatic: they may vary according to the energy consumption of the processes, the logistics of transport, and, above all, the effects on performance and durability, which determine the need for interventions and maintenance over time [2]. For these reasons, the evaluation of the environmental potential of a process demands a life cycle perspective, including construction, maintenance, use, and end-of-life stages, avoiding impact “displacement” between stages (burden shifting) [3].

The approach is aligned with the development of the literature: research in pavement sustainability has been growing and diversifying, with emphasis on both the implementation of new technologies (recycled materials and industrial byproducts) and the adaptation of LCA methodologies to the peculiarities of construction, operation, maintenance, and rehabilitation [1,4,5,6].

Among circular economy strategies with promising prospects in pavements, the use of milled material from asphalt layers (Reclaimed Asphalt Pavement) stands out. RAP is produced in maintenance and rehabilitation activities and is already employed in several countries in asphalt mixtures and as a granular material in base and sub-base layers [7,8,9,10,11]. Data from 2020 indicate that, generally, the main destination of RAP is the production of hot/warm mixtures: in the United States, for example, 90.3% of available RAP was directed to that application, while 6.0% was used in granular layers and 0.4% in cold recycling [12,13]. In Europe, the same trends can be observed in several countries (e.g., Germany 84%, Denmark 85%, France 86%, Finland 100% and Ireland 100% in hot/warm mixtures), while cold recycling is only relevant in some specific contexts (e.g., Slovakia 30%, Czechia 25% and Romania 100%) [12,13]. However, in some countries, RAP is frequently employed in granular pavement layers (63% in the UK, 65% in Norway, 60% in Italy and 98% in Turkey), indicating that, while this is not the main route on a global scale, its structural application as a granular material is still widely employed in different technological contexts [12,13]. In this respect, recent research on mixed reclaimed asphalt materials (MRAMs) has shown that thermal processing, including the use of solar energy, combined with appropriate compaction, can activate the load-bearing capacity of the material by promoting particle bonding and the formation of a cohesive load-bearing structure [14]. This finding further supports the broader technical rationale for thermally conditioned RAP applications in structural pavement layers.

In that scenario, Brazil emerges as an example in which, while institutional incentives exist [15], RAP is still underutilized and not properly tracked: appraisals from concessionaires (2019–2021) indicate a predominance of poorly elaborated destinations (53.2% donations and 37.6% stockpiling), while its use in pavements has a low share (3.1% in base layers and 0.7% in hot/warm mixes) [16]. Furthermore, in contexts where recycling is consolidated, more maturity in the control of and content of the incorporated RAP can be observed: in Japan, the content of RAP in hot/warm recycled mixtures increased from 33% (2000) to 47% (2013) [17,18], and, in the Canadian province of Quebec, 56% of hot/warm mixes produced contain up to 20% RAP. In Brazil, however, only a few concessionaires reported recycling in hot/warm mixes, with an average proportion of around 15% [16]. This scenario reinforces the pertinence of investigating methods with better technological control and higher percentages of RAP.

From a mechanical performance standpoint, the literature indicates that RAP–virgin aggregate blends may exhibit resilient modulus (MR) values exceeding those of unbound virgin aggregate [8,9,10,11,19], indicating enhanced structural stiffness. However, the permanent deformation (PD) response remains less conclusive, with inconsistent findings and pronounced dependence on the stress state, binder content and aging condition, particle size distribution, and compaction and testing protocols [20,21,22,23,24]. The presence of an aged binder film coating RAP particles may promote the accumulation of plastic strains under cyclic loading [25], and repeated load triaxial testing demonstrates the high sensitivity of deformation behavior to stress conditions and mixture composition [26,27,28,29,30]. This response is consistent with the hybrid mechanical nature of RAP, which exhibits characteristics intermediate between granular geomaterials and asphalt-bound composites, thereby imposing additional complexities for constitutive modeling and pavement design. Consequently, design approaches based solely on the resilient response may underestimate rutting susceptibility under specific loading regimes, and the incorporation of permanent deformation as a complementary performance criterion is recommended [11,22,30,31].

To address this complexity, research has investigated the thermal conditioning of RAP as a strategy to enhance its mechanical response when used as an unbound material in structural layers, promoting reduced compressibility and changes in behavior under repeated loading [32,33]. Additional studies demonstrate that compaction temperature and the thermal history of the material significantly influence permanent deformation and resilient modulus measured at ambient temperature, establishing temperature as a critical variable governing both construction procedures and mechanical performance [10,23,34,35,36]. In light of these findings, it is evident that thermal conditions during construction directly affect the mechanical response of RAP, which explains why the literature associates warmer climatic contexts with more favorable performance conditions. More recently, investigations focused on the thermal conditioning of RAP from the perspective of aged binder activation and recycled mixture behavior, although not directly applied to base layer structural solutions, have further reinforced the role of temperature in governing the mechanical response of the material [37].

Collectively, these findings underpin the warm base concept proposed by Coelho et al. [38,39], defined as the application of RAP in base layers following controlled thermal conditioning to enhance compaction and partially mobilize the contribution of the residual binder, thereby expanding its applicability for structural purposes. Nevertheless, the warm base concept incorporates a recurring point of concern: the additional energy demand associated with heating, which may raise questions regarding the net environmental benefit of the solution. Consequently, framing the warm base as a sustainable alternative, or, more precisely, as an alternative with environmental potential, requires quantifying, from a life cycle perspective, the trade-off between process-related energy consumption, the reduction in virgin material inputs, and indirect effects on durability and maintenance. In this context, life cycle assessment (LCA) provides an appropriate methodological framework for evaluating such trade-offs in a consistent and transparent manner.

In this context, eco-efficiency-based approaches provide a structured pathway for integrating performance and environmental assessment by relating the “value” delivered by the pavement system (e.g., service life) to the environmental burdens quantified through LCA [40,41,42,43,44]. Operationally, this enables the comparison of alternatives not solely on a cradle-to-gate impact basis, but also in terms of their capacity to sustain the required level of service over the analysis horizon, incorporating maintenance interventions and their associated environmental impacts.

Although this discussion is applicable to pavement engineering in general, the airport context represents a more critical application scenario, characterized by high reliability requirements, operational constraints, and the strategic importance of pavement management. As reported by several authors, there remains a scarcity of studies and specific guidelines addressing recycling practices and the use of alternative materials in airport pavements [45,46,47], including RAP. This scenario makes it particularly relevant to assess, in an integrated manner, alternatives capable of reconciling structural performance and life cycle considerations, since durability directly influences the frequency of interventions and, consequently, the associated environmental and operational impacts.

In this context, the central hypothesis of this study is that thermal conditioning of RAP, applied as a warm base without chemical stabilizers, may achieve environmental performance equal to or superior to that of binder-stabilized alternatives when evaluated up to the construction stage, while maintaining equivalent structural functionality in terms of service life. The innovative aspect of this work lies in the integrated evaluation of experimentally calibrated mechanical parameters, mechanistic–empirical structural modeling, and LCA within a unified performance-based framework. By coupling stress-dependent constitutive behavior with environmental assessment, the study provides a quantitative basis for assessing eco-efficiency in recycled pavement base layers under functionally equivalent conditions. Accordingly, this study aims to quantify the environmental potential and eco-efficiency of RAP-based structural alternatives, including the warm base solution, using airport pavements as an applied case study.

## 2. Literature Review

Table 1 synthesizes recent studies published over the past five years with the explicit objective of identifying methodological patterns and research gaps in LCA approaches applied to RAP-based pavement layers. The synthesis emphasizes the impact assessment phase (Life Cycle Impact Assessment-LCIA), system boundaries, functional units, and stabilization strategies, as these dimensions directly influence the comparability and interpretation of environmental results.

Rather than providing a detailed review of intrinsic physical or microstructural material properties, the table organizes the literature according to key methodological parameters that define how environmental performance is quantified. The selected columns reflect the principal analytical dimensions of LCA studies, namely the assessment phase (LCI, LCIA, or full LCA), system boundary definition (e.g., cradle-to-gate or cradle-to-construction), impact assessment method, and base-layer configuration, thereby enabling identification of recurring evaluation patterns and potential gaps.

The literature indicates that, although the use of RAP in base layers has been consistently associated with environmental advantages when evaluated through LCA, such benefits have been achieved almost exclusively through stabilization strategies involving additives, including hydraulic binders (cement, slags, fly ash), asphalt emulsions, recycling agents, polymers, or mechanical reinforcement solutions such as geocells. Among studies that effectively apply LCA to RAP base layers, a recurrent pattern emerges: improvements in structural performance and reductions in environmental impacts are generally dependent on the incorporation of additional materials, which frequently become environmental hotspots during the production phase, particularly under cradle-to-gate or cradle-to-construction scopes.

A closer examination of the studies summarized in Table 1 further clarifies the structural–environmental trade-offs reported in the literature. Compared with conventional non-recycled materials, RAP-based solutions tend to reduce the demand for virgin aggregates and binders, resulting in lower environmental impacts during the production phase, as reported by [48,49,50].

However, when RAP is used without stabilization, its behavior resembles that of unbound granular materials, which may compromise mechanical performance, particularly in terms of permanent deformation and structural stability. To address these limitations, several studies [51,52,53] demonstrate that the incorporation of binders such as asphalt emulsions and cement significantly improves stiffness and structural capacity.

Nevertheless, these improvements are systematically associated with the addition of new materials, which become relevant environmental hotspots during the production stage. Even in analyses adopting broader system boundaries [54,55,56], the environmental performance of RAP-based solutions remains strongly dependent on stabilization strategies and subsequent maintenance interventions. Similarly, mechanical reinforcement approaches [57] and optimization of cementitious systems [58,59] confirm that structural gains are predominantly achieved through material addition, implying increased embodied impacts during the production phase.

Notably, within the scope of the surveyed literature, no studies were identified that explicitly assess RAP base layers stabilized exclusively through controlled thermal conditioning without chemical additives under an LCA framework. Although the warm base technique has previously demonstrated mechanical feasibility in terms of resilient modulus and resistance to permanent deformation, its environmental performance has not yet been systematically evaluated through LCA under functionally equivalent structural conditions. This observation indicates a relevant gap in the literature regarding the integrated environmental assessment of thermally conditioned RAP bases and their comparison with additive-stabilized alternatives based on equivalent structural performance assumptions.

**Table 1:** Overview of recent LCA studies on RAP-based pavement layers.

Article	Pavement Layer	Material	LCA Scope (Main Processes)	System Boundary	LCA Phase	Impact Method
Costa et al. [48]	Asphalt surface layer	Asphalt mixture with RAP	Raw materials; binder/aggregates; mixture production	Cradle-to-gate	LCI + LCIA	IPCC GWP (100 years)
Oreto et al. [49]	Asphalt surface layer	Asphalt mixtures with RAP	Raw materials; binder/aggregates; mixture production; internal transport	Cradle-to-gate	LCI + LCIA	CML 2001
Sarabandi et al. [51]	Base layer	Cold recycled RAP (CRM) + emulsion + additives	Virgin aggregates; RAP milling/processing; emulsion/additives; transport; plant production	Cradle-to-gate	Full LCA	ReCiPe 2016 Endpoint (H)
Moins et al. [54]	Base layer (pavement level)	Lean asphalt base with RAP (38–56%) vs. RCA/CTB options	Materials; RAP/RCA processing; production; transport; construction; demolition; waste; recycling credit	Cradle-to-cradle	Full LCA	ReCiPe 2016 Endpoint (H)
Russo et al. [56]	Binder course and base layer	HMA and CRM with RAP + CDW/JGW/FA	Aggregates/filler; binders; transport; production; construction; EoL	Cradle-to-grave	Full LCA	ReCiPe 2016 Midpoint (H)
Hasan et al. [50]	Asphalt surface layer	Asphalt mixtures with RAP (varying content)	Aggregates; binder; RAP processing; mixture production	Cradle-to-gate	LCI + LCIA	IPCC GWP (100 years)
Xu et al. [53]	Base + underlying layer	CSPM base + CRME underlying layer	Material production; transport; mixture production/transport; construction	Cradle-to-construction	LCI + LCIA	CO_2_-eq + energy (factor-based)
Xia et al. [52]	Base layer (FDR)	FDR with RAP + Portland cement (cold)	Materials; transport; construction	Cradle-to-construction	Simplified LCI + LCIA	CO_2_-eq + energy (factor-based)
Moins et al. [55]	Surface and base layers	HMA with RAP (40–70%) + rejuvenators	Materials; transport; production; construction; rehab (mill & replace)	Cradle-to-grave	LCI + LCIA + LCCA	ReCiPe 2016 (H) Endpoint (SS)
Lei et al. [58]	Not applicable (concrete)	Concrete mixes (cement variations/additions)	Raw materials; cement; concrete production; transport; construction (scenario)	Cradle-to-gate/to-construction	Full LCA (incl. optimization)	ReCiPe 2016 Midpoint (H)
Badiger et al. [59]	Not applicable (cementitious)	Cement/concrete scenarios (optimization)	Raw materials; binder production; material production; transport	Cradle-to-gate	Full LCA	ReCiPe 2016 Midpoint (H)
Khan & Puppala [57]	Base layer	RAP base reinforced with geocell (GRRB)	Materials; transport; base construction	Cradle-to-construction	LCI + simplified assessment	PaLATE (energy + emissions)

## 3. Materials and Methods

This section presents the methodological framework adopted for the comparative environmental and structural assessment of RAP base-course alternatives in airport pavements. The analysis integrates mechanistic–empirical structural modeling with LCA.

**Figure 1:**
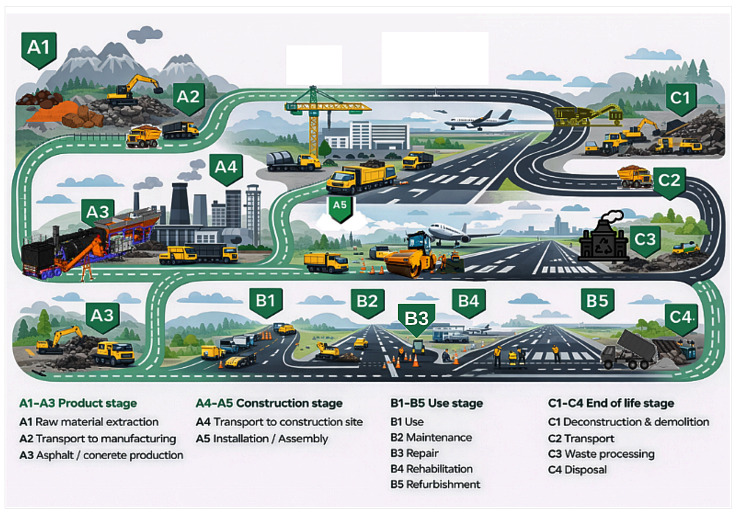
Construction life cycle stages (A1–A5), with A1–A3 and A5 included in the LCA and A4 excluded from the calculations. Adapted from One Click LCA [60]; visually stylized with AI assistance and finalized by the authors in Lucidchart, web-based free plan.

The LCA was conducted under a cradle-to-construction perspective, encompassing raw material supply and production (A1–A3) and construction and installation processes (A5), as illustrated in Figure 1. The transportation stage (A4) was not included in the life cycle calculations, as identical sourcing conditions and transport distances were assumed for all alternatives. Under this assumption, transportation would contribute equally to each scenario and would not influence the comparative results.

As part of the visual preparation of Figure 1, the original reference image from One Click LCA [60] was adapted with the assistance of ChatGPT (OpenAI, GPT-5.4 Thinking) exclusively for visual stylization so that the final scheme better matched the airport pavement context addressed in this study. The final layout and flowchart structure were subsequently reviewed and refined by the authors using Lucidchart. No scientific data, results, or technical interpretations were generated or modified by AI.

**Figure 2:**
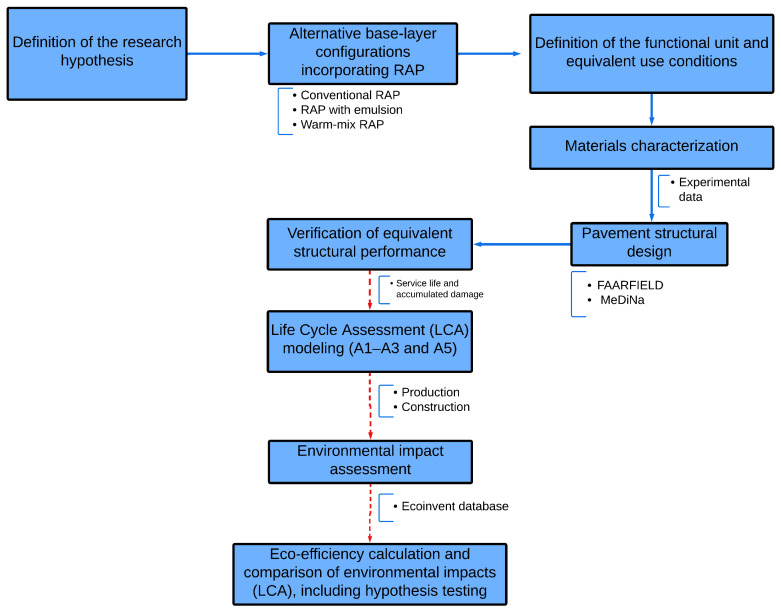
Methodological workflow adopted in this study for the comparative LCA-based assessment (A1–A3 and A5) of RAP base-course alternatives.

The overall methodological workflow adopted in this study is summarized in Figure 2, highlighting the integration of laboratory testing, mechanistic structural modeling, and LCA. The individual stages of this procedure are described in detail in the following subsections.

### 3.1. Pavement Structure and Base Alternatives

A representative airport pavement structure was defined based on geometric, structural, and traffic parameters consistent with typical operational conditions. The reference configuration was established to ensure that all alternatives were evaluated under identical loading, layer configurations, and performance criteria, so that differences in structural and environmental responses could be attributed exclusively to the base-layer solution.

Three base-course alternatives incorporating RAP were analyzed, differing only in their processing and stabilization strategies. Each alternative was designed to fulfill the same structural function within the pavement system, enabling a consistent comparative assessment within the LCA framework.

In the present study, material characterization encompasses the origin, compositional features, and processing conditions of each alternative, as well as the mechanical parameters governing structural response. Detailed physical and mineralogical characterization of the RAP material, including aggregate composition and residual binder properties, has been previously reported in [30,39]. The emphasis herein is placed on the mechanical behavior parameters that directly influence structural performance modeling and subsequent environmental assessment.

#### 3.1.1. Conventional RAP

The conventional RAP, named **RAP 25**, corresponds to the use of RAP as a granular material, without additional thermal treatment or chemical stabilization, as described by Coelho et al. [39]. The RAP was obtained from milling operations on urban pavements in Rio de Janeiro, Brazil, and its physical and mineralogical characteristics were previously reported by the authors. In summary, the material is composed predominantly of quartz-rich crushed aggregates with silicate mineral constituents commonly associated with basaltic-type aggregates used in pavement construction and contains residual asphalt binder typically classified as CAP 30/45.

The material is incorporated directly into the base layer at room temperature (approximately 25 °C), with a moisture content of 5.4%, and its composition was considered to be 100% RAP. Under these conditions, the mechanical behavior of the layer is predominantly associated with that of an unbound granular material.

#### 3.1.2. Warm Base

In the alternative designated as **RAP 110**, the same RAP material as described for RAP 25 is subjected to controlled thermal conditioning prior to compaction, according to the warm base concept proposed by Coelho et al. [39]. The material is heated to approximately 110 °C in order to reach a compaction temperature close to 80 °C. The base composition remains 100% RAP, without the incorporation of any additional materials, including water. The objective of the thermal treatment is to promote partial mobilization of the aged residual asphalt binder, thereby enhancing internal cohesion between aggregate particles. The energy demand associated with the heating process is explicitly accounted for in the life cycle assessment modeling.

#### 3.1.3. Cold Recycled Asphalt Mixture

The third alternative corresponds to a cold recycled asphalt mixture (CRAM) base. In this study, the mixture design and experimental data reported by Coelho et al. [30] were adopted and, for identification throughout the manuscript, this option is known as **RAP + emulsion**. In general, CRAM solutions are composed of RAP, virgin aggregates for gradation adjustment, a bituminous binder (asphalt emulsion or foamed asphalt), and an active filler such as ordinary Portland cement (OPC) or hydrated lime.

In the specific configuration considered herein, RAP is stabilized predominantly through the addition of an RL-1C cationic asphalt emulsion, with CP-II-F-32 Portland cement used as a supplementary hydraulic binder. The adopted mix design comprises 76% RAP, 23% fine aggregate (stone dust), 3% asphalt emulsion, and 1% cement, characterizing a cold-stabilization approach widely documented in the technical literature [61,62,63,64,65]. The additional inputs associated with the emulsion and cement are explicitly accounted for in the life cycle inventory of this alternative.

### 3.2. Mechanical Characterization and Structural Definition of the Pavement

The mechanical behavior of the RAP and granular materials was evaluated through repeated load triaxial (RLT) tests (Owntec equipment), in accordance with DNIT 134 [66] for resilient modulus (RM) and DNIT 179 [67] for permanent deformation (PD). The tests were conducted on cylindrical specimens (100 mm diameter × 200 mm height) under controlled confining and deviator stress states, and axial strains were measured to characterize the stress-dependent response of the materials. All RM and PD parameters correspond to laboratory-controlled RLT conditions at ambient temperature (approximately 25 °C), ensuring that the thermal conditioning applied during construction does not directly influence the constitutive calibration.

The resilient modulus was represented using the composite nonlinear model, as given in Equation (1):

(1) $$RM=K_{1}\;\sigma_{3}^{K_{2}}\;\sigma_{d}^{K_{3}}$$

where RM is the resilient modulus (MPa), $\sigma_{3}$ is the confining stress (kPa), $\sigma_{d}$ is the deviator stress (kPa), and $K_{1}$–$K_{3}$ are regression coefficients obtained from laboratory testing.

The specific permanent deformation was modeled using the empirical expression prescribed by DNIT Standard 179 [67]:

(2) $$\varepsilon_{p}\left(\%\right)=\psi_{1}{\left(\frac{\sigma_{3}}{\rho_{0}}\right)}^{\psi_{2}}{\left(\frac{\sigma_{d}}{\rho_{0}}\right)}^{\psi_{3}}N^{\psi_{4}}$$

where $\varepsilon_{p}$ is the specific permanent deformation (%), $\sigma_{3}$ is the confining stress (kPa), $\sigma_{d}$ is the deviator stress (kPa), $\rho_{0}$ is the reference pressure (kPa), *N* represents the number of load cycles, and $\psi_{1}$–$\psi_{4}$ are regression coefficients obtained from laboratory tests.

The PD parameters were used exclusively to characterize the nonlinear accumulation behavior of unbound and recycled layers within the mechanistic framework implemented in the Brazilian National Pavement Design Method (MeDiNa software, version 1.1.9).

The calibrated regression parameters obtained from the RLT tests for each base alternative are presented in Table 2. The coefficients correspond to the composite resilient modulus model (K_1_–K_3_) and the permanent deformation model ($\psi_{1}$–$\psi_{4}$), together with the respective coefficients of determination (R^2^). The R^2^ values obtained for both models indicate an adequate statistical fit, supporting the reliability of the calibrated parameters for use in the mechanistic analyses.

The structural analysis and design of the airport pavement were carried out using FAARFIELD (version 2.1.1). The software was employed to define the structural sections considered and to establish an aircraft traffic mix representative of the operational conditions considered, based on the annual number of departures and aircraft operational parameters (Table 3).

Based on the estimated cumulative damage factors (CDF) for the considered aircraft fleet, the Boeing B777-300ER was identified as the critical aircraft, based on the maximum CDF value obtained in the FAARFIELD damage accumulation analysis.

An initial structural configuration was analyzed in FAARFIELD using tabulated elastic modulus values to define the aircraft loading conditions and stress levels within the pavement structure. The corresponding stress states were then implemented in the Brazilian National Pavement Design Method (MeDiNa software, version 1.1.9), which operates through the AEMC routine (Elastic Analysis of Multilayer Systems), together with the calibrated composite model parameters (K_1_–K_3_) obtained from the RLT tests.

Within this framework, the nonlinear, stress-dependent behavior of granular and recycled layers was explicitly considered. The AEMC analysis was used to compute the confining and deviator stresses ($\sigma_{3}$, $\sigma_{d}$) at multiple points within each layer of the pavement structure. Based on these results, the critical stress condition was identified, and the corresponding resilient modulus was calculated using Equation (1), resulting in a stress-adjusted equivalent linear modulus for each layer. The representative modulus values reported in Table 4 correspond to the average equivalent modulus obtained under the critical stress condition. These updated linearized modulus values were subsequently reintroduced into FAARFIELD for structural life estimation under aircraft loading.

It is important to emphasize that, although only the base-layer composition was modified among the alternatives, the equivalent modulus values of the overlying and underlying layers also vary. The same calibrated nonlinear material parameters were adopted for all configurations; however, due to the stress-dependent behavior of the granular materials, changes in base stiffness alter the stress distribution within the multilayer system, thereby modifying the confining and deviator stresses acting in each layer. Since the resilient modulus is computed as a function of these stress states, distinct stress-adjusted equivalent linear modulus values are obtained for each structural configuration. This behavior reflects the inherent coupling between layer stiffness and stress redistribution in multilayer elastic systems. The subgrade (natural foundation layer) was modeled as a semi-infinite linear elastic layer; therefore, its modulus remained constant among the analyzed alternatives.

**Figure 3:**
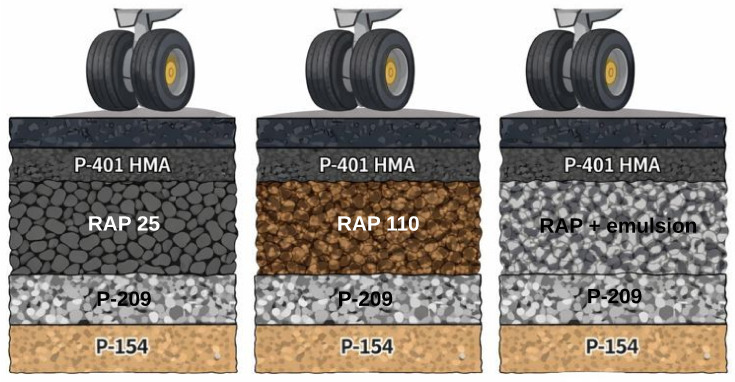
Schematic representation of the analyzed airport pavement structural sections, indicating the base-layer alternatives evaluated.

Figure 3 schematically illustrates the structural sections considered in order to facilitate the visualization of the different base solutions evaluated.

It is important to emphasize that, although only the base-layer composition was modified among the alternatives, the equivalent modulus values of the overlying and underlying layers also vary. The same calibrated nonlinear material parameters were adopted for all configurations; however, due to the stress-dependent behavior of the granular materials, changes in base stiffness alter the stress distribution within the multilayer system, thereby modifying the confining and deviator stresses acting in each layer. Since the resilient modulus is computed as a function of these stress states, distinct stress-adjusted equivalent linear modulus values are obtained for each structural configuration. The subgrade (natural foundation layer) was modeled as a semi-infinite linear elastic layer; therefore, its modulus remained constant among the analyzed alternatives.

### 3.3. Life Cycle Assessment

The LCA was conducted in accordance with the guidelines of ISO 14040 [68] and ISO 14044 [69], comprising the stages of goal and scope definition, life cycle inventory (LCI) analysis, life cycle impact assessment (LCIA), and interpretation of the results.

#### 3.3.1. Objective and Scope

The objective of the LCA was to quantify and compare the environmental impacts associated with different RAP-containing base-layer alternatives under equivalent structural conditions, within the context of airport pavements. The system boundaries were defined according to a cradle-to-construction perspective, encompassing raw material extraction and production (A1–A3) and construction processes (A5). The transportation stage to the construction site (A4) was not included in the life cycle calculations, under the assumption of equivalent logistical conditions for all evaluated alternatives.

The functional unit adopted was 1.0 kg of base material produced and applied, allowing a direct comparison among the evaluated alternatives in terms of environmental impacts per unit of material. This functional unit was selected exclusively for comparative purposes, given that the evaluated alternatives were defined under equivalent structural and construction conditions.

#### 3.3.2. Life Cycle Inventory Analysis and Life Cycle Impact Assessment

LCI analysis was carried out based on secondary data extracted from the Ecoinvent database (version 3.8), complemented by specific data on mixture composition and energy consumption associated with the alternatives analyzed.

The processes considered in the inventory represent the main inputs and activities related to the production and execution of the base layers, including the production of aggregates, bituminous binders, Portland cement, non-ionic surfactant (associated with asphalt emulsion), and thermal energy consumption. The corresponding environmental impact factors for these processes, used as input data in the LCA, are presented in Table 5. In the present study, material transportation was not explicitly included in the life cycle inventory, since the analysis is comparative in nature and assumes equivalent application conditions for all evaluated alternatives.

The LCIA was conducted using the IPCC 2023 method for global warming potential (GWP), the Abiotic Depletion Potential (ADP) for abiotic resource depletion, and the Ecological Scarcity (ES) method, enabling the quantification of the environmental impacts associated with each analyzed alternative. The environmental impact factors adopted as input data for the LCA of the main processes considered in the analysis are presented in Table 5.

In the case of heated RAP, the energy required to raise the material temperature from ambient conditions (25 °C) to 110 °C was estimated. The energy consumption associated with RAP heating was calculated based on specific heat values of asphaltic materials reported in the literature. For this purpose, an average value of 0.92 kJ kg^−1^ K^−1^ was adopted, obtained from experimental data on volumetric heat capacity and density of asphalt mixtures reported by Mirzanamadi et al. [70]. This value was calculated as the ratio between volumetric heat capacity and material density, considering the average of the experimental results reported for different asphalt concrete mixtures.

The energy required to raise the material temperature from ambient conditions (25 °C) to 110 °C was then modeled in the LCA through the thermal energy process presented in Table 5, using the corresponding environmental impact factors. It is emphasized that only experimental values reported in the reference study were used, rather than values obtained from numerical modeling.

The environmental impacts were primarily expressed in terms of carbon dioxide equivalent emissions (CO_2_-eq), allowing a direct comparison among the evaluated alternatives.

#### 3.3.3. Interpretation and Integration with Structural Performance

The interpretation of the results was guided by the concept of eco-efficiency, originally disseminated by Schmidheiny and co-authors and later consolidated by the World Business Council for Sustainable Development (WBCSD) [71,72,73,74]. In the present study, eco-efficiency was operationalized by integrating the environmental impacts estimated through LCA with the structural performance results. In general, eco-efficiency is interpreted as the ecological optimization of production systems, often expressed as the relationship between the value generated and the associated environmental burden. In the context of asphalt pavements, eco-efficiency has been applied as a decision-support tool to compare structural and rehabilitation alternatives by integrating environmental, energy, economic, and performance indicators over the life cycle. Such assessments have been used both for long-life pavements and for maintenance and recycling strategies, demonstrating their suitability for comparative analyses of solutions with different structural configurations and design horizons [41,75].

In this study, eco-efficiency was implemented by integrating structural response outcomes with the environmental impacts estimated by LCA, following an approach in which impacts are normalized as a function of performance and structural service life, consistent with methodologies previously applied in transport infrastructure studies [76]. The structural service life obtained in FAARFIELD was adopted as the indicator of technical value, while the LCA impacts represented the environmental dimension of the analysis. This integration enables a comparative assessment of each pavement alternative in terms of its ability to deliver greater structural performance over its service life with lower environmental impact.

## 4. Results and Discussion

### 4.1. Structural Performance and Estimated Service Life

Based on the mechanical parameters of the materials and the adopted aircraft mix, the structural service life and the cumulative damage contributions for the analyzed pavements were estimated. Table 6 presents the estimated service life values and the cumulative damage factors associated with the considered structural layers.

The results indicate significant differences in structural performance among the evaluated alternatives. The pavement with a warm RAP base exhibited the highest structural service life, associated with the lowest cumulative damage factors in both the subbase and the asphalt layer.

In quantitative terms, the estimated service life for RAP 110 was approximately 270% greater than that of the RAP 25 pavement and about 370% greater than that of the RAP + emulsion alternative. Conversely, the RAP + emulsion solution presented a service life approximately 21% lower than that of RAP 25, indicating that, under the evaluated conditions, the stabilization effect did not result in substantial structural gains. It should be noted that this result reflects the specific mechanical response of the cold recycled mixture adopted in this study and should not be interpreted as a generalized behavior of emulsion-treated RAP bases. Nevertheless, it highlights that emulsion stabilization does not necessarily lead to superior structural performance, reinforcing the importance of material-specific evaluation when compared with alternative techniques such as warm RAP bases.

The superior performance observed for heated RAP can be attributed to the improvement in the mechanical behavior of the base layer, associated with greater activation of the aged binder and increased internal cohesion of the mixture [77]. Unlike cold-recycled or emulsion-stabilized mixtures, in which particle bonding occurs predominantly through localized binder connections, RAP heating promotes the formation of a more continuous and mechanically efficient structure, resulting in a more stable response under repeated loading [39,77].

This behavior is consistent with observations reported in the literature, according to which heated RAP mixtures exhibit lower susceptibility to permanent deformation and lower damage accumulation rates than cold-recycled mixtures, as evidenced by multi-stage mechanistic tests [38]. In contrast, studies indicate that, although the addition of emulsion or stabilizing agents improves the initial performance of recycled mixtures, this mechanism is not sufficient to eliminate the progressive accumulation of deformations under repeated loading [30], which explains the more modest differences observed between the non-heated RAP and emulsion-stabilized RAP alternatives.

The environmental assessment of the materials was carried out based on life cycle inventory data, considering the IPCC 2023, ADP, and ecological scarcity indicators. Table 7 presents the estimated environmental impacts for each analyzed alternative.

The results indicate that heated RAP presents intermediate environmental impacts when compared to the other analyzed alternatives. This behavior is associated with the additional energy consumption required for heating the material, resulting in higher emissions than those observed for non-heated RAP. On the other hand, the RAP stabilized with emulsion exhibited the highest environmental impacts, reflecting the additional contribution of processes related to binder and stabilizing agent production. Similar trends have been reported in LCA studies applied to recycled asphalt mixtures [52,54,78].

Comparable findings have also been reported in the literature, indicating that the incorporation of recycled materials into asphalt base layers can reduce environmental impacts relative to conventional solutions. However, the introduction of additional processes, such as heating or chemical stabilization, tends to increase environmental burdens during the production phase [51,54]. From a mechanical standpoint, although recycling and stabilization techniques contribute to mitigating permanent deformation, these mixtures may still exhibit cumulative deformation over time, particularly under repeated loading, as indicated in consolidated technical guidelines and recent experimental studies [30,79]. In this context, structural service life plays a fundamental role in the sustainability assessment of the analyzed alternatives.

### 4.2. Eco-Efficiency Analysis

The eco-efficiency of the analyzed alternatives was evaluated by normalizing the environmental impacts with respect to the estimated structural service life (SSL). For this purpose, an eco-efficiency indicator was adopted, defined as the ratio between the environmental impact associated with greenhouse gas emissions (IPCC 2023, in kg CO_2_-eq) and the estimated structural service life for each alternative, as expressed in Equation (3).

(3) $$EE=\frac{\mathrm{EII}}{SSL}$$

where:

EE Eco-efficiency indicator (kg CO_2_-eq · year^−1^);
EII Environmental impact indicator (kg CO_2_-eq);
SSL Structural service life (years).

Table 8 presents the eco-efficiency indicator values obtained for the analyzed alternatives, enabling a direct comparison of environmental impacts normalized by structural performance over the service period.

The eco-efficiency analysis results indicate that, although heated RAP presents intermediate absolute environmental impacts, this alternative exhibits the lowest environmental impact when normalized by structural service life. As shown in Table 8, the IPCC 2023 impact per year of service estimated for RAP 110 is approximately 3.5 times lower than that of untreated RAP and nearly one order of magnitude lower than that of the emulsion-stabilized RAP alternative.

However, when environmental impacts are analyzed jointly with structural performance and estimated service life, the mechanical superiority of the warm base plays a decisive role in reducing environmental impacts over the service period. The significantly longer service life of this solution enables the initial environmental burdens to be distributed over time, reducing the need for future interventions and the impacts associated with maintenance activities, in line with integrated approaches combining LCA and mechanical performance reported in the literature [23,33,51,80]. Previous studies comparing hot- and cold-mix asphalt mixtures containing RAP in base layers have likewise indicated that solutions with enhanced mechanical performance may achieve environmental advantages when assessed from a life cycle perspective, despite requiring higher energy consumption during the production phase [54,80].

## 5. Conclusions

This study evaluated the structural performance and environmental impacts of different base-layer solutions containing Reclaimed Asphalt Pavement (RAP), with a focus on the application of heated RAP in airport pavements. The analysis was conducted through an integrated approach, combining mechanistic–empirical modeling of structural performance with a Life Cycle Assessment (LCA) covering material production (A1–A3) and construction processes (A5), while excluding transportation (A4) under equivalent logistical assumptions. The aim was to investigate the eco-efficiency of the alternatives under functionally equivalent service conditions. The results demonstrated significant differences among the analyzed solutions, which can be summarized as follows:

- The heated RAP (RAP 110) solution presented the highest estimated structural service life (58.9 years), approximately 270% higher than that of RAP 25 (15.9 years) and 370% higher than that of RAP + emulsion (12.5 years). This performance was associated with substantially lower cumulative damage factors in both the subbase (CDF = 0.28) and the HMA layer (CDF = 0.17), indicating a more stable mechanical response under repeated aircraft loading.
- In terms of environmental impacts within the adopted production and construction scope, RAP 110 exhibited an intermediate global warming potential (0.00739 kg CO_2_-eq), slightly higher than that of RAP 25 (0.00688 kg CO_2_-eq) due to the additional heating energy, but substantially lower than that of RAP + emulsion (0.01551 kg CO_2_-eq). Similar trends were observed for the ADP and ecological scarcity indicators.
- When structural performance and environmental impact were evaluated jointly through the eco-efficiency indicator (IPCC/service life), RAP 110 presented the lowest normalized impact ($1.26\times 10^{-4}$ kg CO_2_-eq · year^−1^), approximately 3.5 times lower than that of RAP 25 and nearly one order of magnitude lower than that of RAP + emulsion. These results indicate that the extended service life of the heated RAP base offsets the moderate increase in production-related emissions, resulting in the most favorable balance between structural performance and environmental impact among the analyzed alternatives.

These results support the research hypothesis that the application of heated RAP in base layers may achieve environmental performance equal to or superior to that of conventional or binder-stabilized solutions within the defined production and construction scope. The findings indicate that construction strategies capable of improving the mechanical response of the base layer can significantly influence the overall sustainability of airport pavements, suggesting that heated RAP (warm base) represents a technically viable and environmentally competitive alternative under the adopted system boundaries. These findings further suggest that construction techniques such as thermal conditioning, which may initially appear less sustainable due to additional energy demand, can exhibit significant environmental potential when evaluated in conjunction with structural performance and service life. This highlights the importance of performance-based sustainability assessments in identifying solutions whose long-term benefits offset moderate increases in production-stage impacts.

Future research adopting an extended life cycle perspective and integrating structural performance modeling with rehabilitation scenarios is recommended to further assess the long-term sustainability of heated RAP base solutions. Although evaluated here within the airport context, the proposed analytical framework may be extended to other pavement applications.

## Figures and Tables

**Table 2:** Calibrated regression parameters for the RM and PD models obtained from RLT testing.

Material	PD Model					RM Model			
	$\psi_{1}$	$\psi_{2}$	$\psi_{3}$	$\psi_{4}$	R^2^	$K_{1}$	$K_{2}$	$K_{3}$	R^2^
RAP 25	0.11202	0.247884	0.53763	0.14784	0.989182	2048.47	0.609701	0.046743	0.989182
RAP 110	0.120929	0.160214	0.551942	0.088312	0.863171	3586.524	0.411913	0.006239	0.863171
RAP + emulsion	0.276702	0.077229	1.196111	0.131912	0.940194	3666.456	0.593484	0.034915	0.885892

**Table 3:** Aircraft mix adopted as input data for the FAARFIELD analyses.

Aircraft	GTW (kg)	Annual Growth Rate	Growth	Annual Departures	CDF	Maximum CDF	P/C
A320-200 std	73,900	7063	1.13	157,222	0.00	0.00	1.08
A321-100 std	83,400	5679	1.13	126,415	0.00	0.00	1.08
B767-300	163,747	5301	1.13	118,000	0.00	0.00	1.06
Bombardier CL-604/605	21,863	3495	1.13	77,799	0.00	0.00	1.22
B777-300 ER	352,441	3056	1.13	68,027	0.89	0.89	1.04
B787-9	255,372	2803	1.13	62,396	0.01	0.01	1.03
B777-200	248,120	3799	1.13	84,566	0.00	0.00	1.04
A330-300 WW20	230,900	842	1.13	18,743	0.00	0.00	1.03
B787-8	228,383	579	1.13	12,889	0.00	0.00	1.04
A310-300	142,900	772	1.13	17,185	0.00	0.00	1.08
B747-400	397,801	245	1.13	5454	0.00	0.00	1.06
B747-400 Belly	397,801	245	1.13	5454	0.00	0.00	1.06
A380-800 WW00	562,000	105	1.13	2337	0.00	0.00	1.04
A380-800 WW00 Belly	562,000	105	1.13	2337	0.00	0.00	1.03
DHC-7	19,867	30,830	1.13	686,276	0.00	0.00	1.22
B737-800	79,242	19,706	1.13	438,656	0.00	0.00	1.09
CRJ100/200	21,636	8552	1.13	190,368	0.00	0.00	1.21

**Table 4:** Mechanical properties of the pavement layers for each base-course alternative.

Layer	Thickness (mm)	Modulus (MPa)		
		RAP 25	RAP 110	RAP + Emulsion
P-401 HMA Surface	304	1778.32	1543.23	1803.69
P-209 Crushed aggregate	300	185.75	741.05	151.36
P-154 Uncrushed aggregate	900	163.83	163.20	163.39
Subgrade	–	51.71		

**Table 5:** Environmental impact indicators considered in the LCA.

Process	IPCC 2023	ADP	Ecological	Ecoinvent
	(kg CO_2_-eq)	(kg Sb-eq)	Scarcity	
Gravel production	0.00505	$2.85\times 10^{-8}$	13.8	gravel production, crushed–BR–gravel, crushed
Pitch production	0.0417	$1.55\times 10^{-7}$	586	pitch production, petroleum refinery operation–BR–pitch
Non-ionic surfactant	4.01	$3.96\times 10^{-5}$	14,200	market for non-ionic surfactant–GLO–non-ionic surfactant
Heat	0.00513	$8.75\times 10^{-8}$	5.63	market for heat, future–GLO–heat, future
Cement production	0.837	$3.00\times 10^{-6}$	631	cement production, Portland–BR–cement, Portland

**Table 6:** Structural service life and cumulative damage factors for the analyzed pavements.

Pavement Alternative	Service Life (years)	Subbase-Layer CDF	HMA CDF
RAP 25	15.9	1.29	5.08
RAP 110	58.9	0.28	0.17
RAP + emulsion	12.5	1.66	6.72

**Table 7:** Estimated environmental impacts for the materials analyzed.

Material	IPCC 2023	ADP	Ecological Scarcity
	(kg CO_2_-eq)	(kg Sb-eq)	(-)
RAP 25	0.0068825	$3.48\times 10^{-8}$	42.41
RAP 110	0.0073944	$4.36\times 10^{-8}$	42.97
RAP + emulsion	0.0155076	$7.30\times 10^{-8}$	44.51

**Table 8:** Eco-efficiency indicator based on the ratio between environmental impact and structural service life.

Pavement Alternative	IPCC 2023	Service Life	IPCC/Service Life	ADP	ADP/Service Life	ES	ES/Service Life
	(kg CO_2_-eq)	(years)	(kg CO_2_-eq · year^−1^)	(kg Sb-eq)	(kg Sb-eq · year^−1^)	(-)	(-year^−1^)
RAP 25	0.0068825	15.9	$4.33\times 10^{-4}$	$3.59\times 10^{-8}$	$2.19\times 10^{-9}$	42.41	2.67
RAP 110	0.0073944	58.9	$1.26\times 10^{-4}$	$1.35\times 10^{-9}$	$7.40\times 10^{-10}$	42.97	0.73
RAP + emulsion	0.0155076	12.5	$1.24\times 10^{-3}$	$2.18\times 10^{-8}$	$5.84\times 10^{-9}$	44.51	3.56

## Data Availability

The original contributions presented in this study are included in the article. Further inquiries can be directed to the corresponding author.
